# Assessment of Lupus Anticoagulant Positivity in Patients With Coronavirus Disease 2019 (COVID-19)

**DOI:** 10.1001/jamanetworkopen.2020.17539

**Published:** 2020-08-12

**Authors:** Morayma Reyes Gil, Mohammad Barouqa, James Szymanski, Jesus D. Gonzalez-Lugo, Shafia Rahman, Henny H. Billett

**Affiliations:** 1 Montefiore Medical Center, Department of Pathology, Albert Einstein College of Medicine, The Bronx, New York; 2 Montefiore Medical Center, Division of Hematology, Department of Medical Oncology, Albert Einstein College of Medicine, The Bronx, New York; 3 Montefiore Medical Center, Department of Medicine, Albert Einstein College of Medicine, The Bronx, New York

## Abstract

This cohort study assesses the prevalence of lupus anticoagulant (LA) positivity in patients with coronavirus disease 2019 (COVID-19) and examines the association of LA positivity with patient outcomes.

## Introduction

Antiphospholipid syndrome (APS) is an autoimmune disease that manifests as arterial, venous, or microcirculatory thromboses as well as obstetric complications.^1^ The diagnosis of APS requires the presence of IgG or IgM anti-β2-glycoprotein-1 or anticardiolipin antibodies by enzyme-linked immunosorbent assay or lupus anticoagulant (LA) assays that must persist for more than 12 weeks. Coagulopathy in patients with coronavirus disease 2019 (COVID-19) is a common complication that jeopardizes the clinical course and is associated with poorer outcomes.^2,3^ This COVID-19 coagulopathy presents mainly as a prothrombotic state, and there is evidence that anticoagulation may reduce mortality rates.^4^ The partial thromboplastin time (PTT) has been found to be prolonged in many patients with COVID-19 and may indicate the presence of LA.^5^ Most patients with COVID-19 have elevated levels of C-reactive protein (CRP), and CRP is known to interfere with LA PTT-based tests, such as the hexagonal phase phospholipid neutralization assay STACLOT-LA.^6^

## Methods

This cohort study was approved by the Albert Einstein College of Medicine institutional review board. We performed a retrospective study to understand the demographic, laboratory, and clinical characteristics of LA-positive patients with COVID-19 (see study design and methods in the eAppendix in the Supplement). LA positivity was determined by the dilute Russell viper venom time (DRVVT). Most samples were also screened for STACLOT-LA and LA serological testing. Statistical methods to study association of LA with outcomes included Fisher exact tests for categorical values, 2-sample *t*-tests for parametric values, Kruskal-Wallis tests for nonparametric values, and logistic regression of DRVVT adjusted by CRP. The threshold for statistical significance was set at 2-tailed *P* < .05. R statistical software version 3.6.2 (R Project for Statistical Computing) was used for analysis.

## Results

One hundred eighty-seven patients had LA testing ordered from March 1 to April 30, 2020, at Montefiore Medical Center, a large tertiary center in the Bronx, New York. Of those, 119 were not tested or tested negative by COVID reverse transcriptase–polymerase chain reaction. The LA-positive rate by DRVVT in patients who tested negative for COVID-19 was 22% (27 of 119). In contrast, the LA-positive rate in patients who tested positive for COVID-19 was 44% (30 of 68) (*P* = .002). Of the 30 COVID-19–positive patients who had a positive LA by DRVVT, 17 (59%) were also positive by hexagonal phospholipid neutralization STACLOT-LA test (Table 1). Eleven (16%) were positive only for STACLOT-LA and those had significantly higher CRP levels. Mean prothrombin time and PTT were more prolonged in LA-positive compared with LA-negative patients (Table 1). Importantly, of the 30 patients who were LA positive, 19 had documented thrombosis (arterial and venous), an event rate of 63%, as compared with a rate for LA-negative patients of 34% (*P* = .03). No statistically significant difference was found by gender, race, ethnicity, ventilation, mortality, and anticoagulation at the time of thrombosis between patients who tested LA positive vs negative (Table 1). Although the mean CRP level was higher in patients testing positive for LA by DRVVT (14.4 vs 7.5 mg/dL; *P* < .01), patients with thromboses did not have significantly higher CRP levels than those with no thromboses (Table 2). After adjusting for CRP, LA was found to be independently associated with thrombosis (odds ratio, 4.39; 95% CI, 1.45-14.57; *P* = .01). Although D-dimer (dimerized plasmin fragment D) on admission was not different between patients with and without LA, initial D-dimer was significantly higher in patients who developed a thrombotic event compared with patients without clots (9.14 vs 4.98 μg/mL; *P* = .04) (Table 2). Only 1 patient among those positive for LA by DRVVT had IgM antibodies against β2-glycoprotein-1 and cardiolipin. The rest were negative for IgM and IgG antibodies against both β2-glycoprotein-1 and cardiolipin.

**Table 1.** COVID-19–Positive Patients: Outcomes in Patients by LA Result^a^

Patient characteristic	No. (%)		*P* value^b^
	LA Negative	LA Positive	
No.	38	30	
Female	20 (52.6)	14 (46.7)	.81
Race			
Black	14 (36.8)	9 (30.0)	.95
White	6 (15.8)	5 (16.7)	
Other	17 (44.7)	15 (50.0)	
Asian	1 (2.6)	1 (3.3)	
Ethnicity			
Hispanic	16 (42.1)	13 (43.3)	.46
Non-Hispanic	21 (55.3)	14 (46.7)	
Declined to state	1 (2.6)	3 (10.0)	
Age, mean (SD), y	50.45 (20.19)	64.77 (13.84)	.001
Admission	35 (92.1)	30 (100.0)	.25
Invasive oxygen	4 (10.5)	5 (16.6)	.49
Mechanical ventilation	19 (50.0)	15 (50.0)	>.99
Mortality	10 (26.3)	14 (46.7)	.12
Coagulation data			
Prothrombin time, mean (SD), s^c^	15.49 (2.63)	19.89 (11.01)	.03
PTT, mean (SD), s^d^	41.13 (18.11)	57.76 (33.72)	.02
Positive STA-CLOT LA, No. positive/No. tested (%)	11/29 (37.9)	17/29 (58.6)	.19
DRVVT			
Normalized ratio, mean (SD)^e^	1.10 (0.08)	1.43 (0.24)	<.001
Mix ratio, median (IQR)	1.18 (1.12-1.23)	1.41 (1.23-1.71)	.001
Confirm ratio, median (IQR)	1.27 (1.15-1.69)	1.52 (1.28-1.88)	.07
Ratio, mean (SD)	1.47 (0.36)	2.66 (1.50)	<.001
ACL, positive/tested (%)			
IgG antibody	0/34	0/28	>.99
IgM antibody	0/34	1/28 (3.6)	.45
β2-glycoprotein-1, No. positive/No. tested (%)			
IgG	0/35	0/27	>.99
IgM	0/33	1/27 (3.7)	.45
Prior APS	0	1 (3.3)	.44
CRP, mean (SD), mg/dL	7.49 (6.04)	14.43 (11.79)	.004
Baseline D-dimer, mean (SD), μg/mL fibrinogen equivalent units	5.74 (6.64)	7.97 (7.76)	.27
D-dimer at thrombotic event, mean (SD), μg/mL	9.53 (6.68)	11.81 (6.95)	.38
Thrombosis	13 (34.2)	19 (63.3)	
Deep venous thrombosis	9 (69.2)	8 (42.1)	.03
Pulmonary embolus	4 (30.8)	3 (15.7)	
Arterial thrombosis	0	6 (31.6)	
Stroke	0	2 (10.5)	
Anticoagulation			
None	21	9	.11
Prophylactic	7	8	
Therapeutic	10	13	

Abbreviations: ACL, anticardiolipin; APS, antiphospholipid syndrome; CRP, C-reactive protein; D-dimer, dimerized plasmin fragment D; DRVVT, dilute Russell viper venom time; IQR, interquartile range; LA, lupus anticoagulant; PTT, partial thromboplastin time.

SI conversions: To convert CRP to mg/L, multiply by 10; D-dimer to nmol/L, multiply by 5.476.

^a^ Positivity for LA was determined by DRVVT.

^b^ *P* value measured by Fisher exact test for all nominal variables owing to the small sample size, *t* test for parametric (mean), and Kruskal-Wallis test for nonparametric values (median).

^c^ Reference range, 11.8 to 14.8.

^d^ Reference range, 25.9 to 38.9.

^e^ Reference range, less than 1.2.

**Table 2.** Thrombotic Events in Study Population

Patient characteristic	No. (%)		*P* value
	No thrombotic event	Thrombotic event	
No.	36	32	
Female	19 (52.8)	15 (46.9)	.81
Mortality	15 (41.6)	9 (28.1)	.31
Age, mean (SD), y	53.1 (21.7)	60.9 (14.5)	.09
CRP, mean (SD), mg/dL	10.95 (10.62)	10.62 (8.99)	.89
First D-dimer, mean (SD), μg/mL fibrinogen equivalent units	4.98 (6.33)	9.14 (7.58)	.04
APS laboratory data, No. positive/No. tested (%)			
Positive STA-CLOT	15/30 (50.0)	13/28 (46.4)	>.99
Positive DRVVT	11/36 (44.8)	19/32 (59.4)	.03
ACL			
IgG antibody	0/32	0/30	>.99
IgM antibody	0/32	1/30 (3.3)	.48
β2-glycoprotein-1			
IgG	0/32	0/32	>.99
IgM	0/30	1/30 (3.3)	>.99
Prior APS	0	1 (3.1)	>.99
Clot type			
Deep vein thrombosis	0	16 (50.0)	NA
Pulmonary embolus	0	8 (25.0)	
Arterial thrombosis	0	6 (18.8)	
Stroke	0	2 (6.2)	
Anticoagulation			
None	16 (44.4)	14 (43.8)	>.99
Prophylactic	8 (22.2)	7 (21.9)	
Heparin	4	2	
Enoxaparin	4	2	
Apixaban	0	3	
Therapeutic	12 (33.3)	11 (34.4)	
Heparin	1	2	
Enoxaparin	2	0	
Apixaban	2	6	
Bivalirudin	6	3	
Warfarin	1	0	
Aspirin	5 (29.4)	8 (25.0)	.75

Abbreviations: ACL, anticardiolipin; APS, antiphospholipid syndrome; CRP, C-reactive protein; D-dimer, dimerized plasmin fragment D; DRVVT, dilute Russell viper venom time; NA, not applicable.

SI conversions: To convert CRP to mg/L, multiply by 10; D-dimer to nmol/L, multiply by 5.476.

## Discussion

This cohort study found an increased incidence of LA positivity in patients with COVID-19 after adjusting for CRP levels. In addition, LA was associated with incidence of thrombosis in patients with COVID-19. Limitations of this retrospective study include a small sample size and inability to control time of LA testing from admission to outcome (mortality and thrombosis). LA-positive individuals have a marked risk of arterial and venous thrombosis, and therapeutic anticoagulation should be considered in these patients.

## Notes

Supplement. **eAppendix.** Additional Information on Study Methods
