# Supplementary material for: Assessment of Lupus Anticoagulant Positivity in Patients With Coronavirus Disease 2019 (COVID-19)
Source: JAMA Netw Open. 2020 Aug 12;3(8):e2017539. doi: 10.1001/jamanetworkopen.2020.17539 (PMC12124689; doi:10.1001/jamanetworkopen.2020.17539)
Supplement: Supplement. — eAppendix. Additional Information on Study Methods [file jamanetwopen-e2017539-s001.pdf]

Supplementary Online Content

Reyes Gil M, Barouqa M, Szymanski J, Gonzalez-Lugo JD, Rahman S, Billett HH. Assessment of lupus anticoagulant positivity in patients with coronavirus disease 2019 (COVID-19). *JAMA Netw Open*. 2020;3(8):e2017539. doi:10.1001/jamanetworkopen.2020.17539

eAppendix. Additional Information on Study Methods

This supplementary material has been provided by the authors to give readers additional information about their work.

© 2020 Reyes Gil M et al. *JAMA Network Open.*

eAppendix

Study Design:

This retrospective study was approved by the Albert Einstein College of Medicine Institutional Review Board. In order to study the incidence of lupus anticoagulants in COVID positive patients, we queried our laboratory information system Epic Beaker, for all patients who had Lupus anticoagulant test ordered between March 1st to April 30th 2020 at Montefiore Medical Center/University Hospital for Albert Einstein College of Medicine. We then sorted the cases that were confirmed COVID positive by reverse-transcriptase–polymerase-chain-reaction real-time (RT-PCR) assay of the nasal and the pharyngeal swabs. Patients who had LA test ordered and who were negative by COVID RT-PCR test, or who did not get COVID testing were considered COVID negative patients. We excluded the patients younger than 18 years of age or who were on bivalirudin or any other direct thrombin inhibitor at time of sample collection for LA testing. The medical record of COVID positive patients was reviewed to obtain the epidemiological, demographic, clinical and laboratory data.

These variables included demographic attributes (age, sex and self-reported race and/or ethnicity) and laboratory data. D-Dimer in the first 48 hrs. of presentation to ED and/or hospital admission was noted. Closest D-Dimer, at least within 36 hrs., to documented clot was also noted. Closest C-Reactive Protein (CRP) to LA testing date was recorded. Thrombosis, date of thrombosis and type of thrombosis was documented as positive based on imaging studies (Doppler ultrasonography or Computer Tomography Angiography). History of prior LA testing was also recorded.

We assessed for interventions and time to interventions including supplemental oxygen, mechanical ventilation and anticoagulation. Anticoagulation treatment at the time of clot detection was recorded, in cases were no clot was detected, anticoagulation was recorded based on the agent the patient received prior to the imaging study, and if no imaging was performed, the anticoagulation prior to the DRVVT test was recorded. Anticoagulation therapy was classified as none, prophylactic and therapeutic. Patients that received anticoagulation for <48hrs. prior to the clot, imaging study or DRVVT test were considered as having received no anticoagulation. Prophylactic doses were: 2.5 mg twice a day for apixaban, 5000 Units every 8 or 12 hours subcutaneously for heparin, 40 mg subcutaneous daily for enoxaparin. Therapeutic doses were: 5 mg twice a day or 10 mg twice a day for apixaban, as well as apixaban 2.5 mg twice a day in patients with CrCl <30 mL/minute, doses ≥ 1.5

© 2020 Reyes Gil M et al. *JAMA Network Open.*

mg/kg daily of enoxaparin, any dose of warfarin with therapeutic INR, and patients on continuous drips of unfractioned heparin or bivalirudin. Discharge from hospital and mortality were assessed.

This report follows the STROBE reporting guideline for observational cohort studies including details of eligibility and exclusion criteria, methods of data collection, definition of exposures and outcome and discussion of potential confounders and effect modifiers.

As chart review was an imminent component of this study data was not deidentified initially but securely encrypted during the chart review process. After chart reviews were completed data were deidentified.

Methodology for coagulation tests:

Coagulation tests (D-Dimer, PT, PTT, DRVVT and STA-CLOT-LA) were performed on same samples submitted for LA testing by STA-R Max instruments using STAGO reagents as per manufacturer recommendations. As many patients were on Direct Oral Anticoagulants (DOAC), DOACs were removed using DOAC-remove (Aniara, OH, USA) on all plasma samples prior to testing as per manufacturer recommendations. Dilute Russell Viper Venom Time (DRVVT) results were reported and interpreted according to the International Society Thrombosis and Hemostasis guidelines. DRVVT reagents contain heparin neutralizers eliminating possible interference from heparin. All samples were screened with the DRVVT assay and a lupus sensitive PTT. Mix studies were performed on all DRVVT tests. Most samples were also tested with the STACLOT-LA assay. The cut off for LA positive (DRVVT and STACLOT-LA) was set up at the 99th percentile of the normal population. Interpretation of LA positivity was based on a normalized ratio $\geq$ 1.2 after eliminating potential drug interferences and taking into consideration the mix result to exclude factor deficiency as the main cause of prolonged DRVVT clotting time.

Statistical Methods:

The medical record of the patients was reviewed to obtain the epidemiological, demographic, clinical and laboratory data. Data analysis was performed using R software, version 3.6.2. Differences in demographic, clinical variables and laboratory assessments between patients who tested positive vs. negative for DRVVT and patients with clots vs. without clots were done using Fisher's exact test for all nominal variables due to the

© 2020 Reyes Gil M et al. *JAMA Network Open.*

small sample size, *t*-test for parametric, Kruskal–Wallis for nonparametric data. Logistic regression was carried out to examine the relationship between the CRP and DRVVT among patients who clotted with confidence interval (CI) of 95%. P value for all statistical methods was considered significant if <0.05.

© 2020 Reyes Gil M et al. *JAMA Network Open.*
